# A path analysis study of factors influencing hospital staff perceptions of quality of care factors associated with patient satisfaction and patient experience

**DOI:** 10.1186/s12913-017-2718-x

**Published:** 2017-11-16

**Authors:** Sandra G. Leggat, Leila Karimi, Timothy Bartram

**Affiliations:** La Trobe University, Bundoora, Victoria 3086 Australia

## Abstract

**Background:**

Hospital staff are interested in information on patient satisfaction and patient experience that can help them improve quality of care. Staff perceptions of quality of care have been identified as useful proxies when patient data are not available. This study explores the organizational factors and staff attitudes that influence staff perceptions of the quality of the care they provide in relation to patient satisfaction and patient experience.

**Methods:**

Cross sectional survey completed by 258 staff of a large multi-campus, integrated metropolitan hospital in Australia. Structured equation modelling was used to analyse the data.

**Results:**

Our data suggest that different perceived organizational factors and staff attitudes contribute to different pathways for patient satisfaction and patient experience indicators. Hospital staff in our sample were more likely to indicate they provided the care that would result in higher patient satisfaction if they felt empowered within a psychologically safe environment. Conversely their views on patient experience were related to their commitment towards their hospital. There was no relationship between the staff perceptions of patient satisfaction and the staff response to the friends and family test.

**Conclusions:**

This study provides empirical evidence that staff perceptions of the quality of care they provide that is seen to be related to patient satisfaction and patient experience are enacted through different pathways that reflect differing perceptions of organizational factors and workplace psychological attitudes.

**Electronic supplementary material:**

The online version of this article (10.1186/s12913-017-2718-x) contains supplementary material, which is available to authorized users.

## Background

Managers and management researchers are interested in healthcare management practices that are linked to improvements in quality of care. There is strong evidence that effective management practices are positively related to staff perspectives on the quality of care provided in their organization [1–5], and that staff perspectives are useful indicators of the quality of care that is provided [5–8]. There is increasing evidence that the satisfaction of consumers of health care is related to the processes of care [9]. The literature focuses on both patient satisfaction and patient experience as representing quality of care [10], and this is the first study to explore hospital staff perceptions using the indicators that hospitals use to measure both patient satisfaction and patient experience. Quality of patient care is the responsibility of all workers within a health service [11, 12] and therefore, unlike previous studies focusing on clinicians, we capture the perspectives of all hospital staff.

The study objective is to identify the organizational factors and staff attitudes that contribute to hospital staff perceptions of the quality of care patients receive in their hospital, measured as patient satisfaction and patient experience. We use cross-sectional survey data collected from a large multi-campus Australian public hospital in 2014, building on previous work by using structural equation modelling (SEM) to explore these relationships.

### Indicators of quality of care

The measurement of quality of care has not been easy in healthcare management studies. It is often difficult to get patient level quality of care data that can be meaningfully correlated with organizational and staff psychological constructs and attitudes. To overcome this, staff perceptions of the quality of patient care have been shown to act as a useful proxy for patient level indicators [5–8, 13]. There were two quality of care variables used in this study. The first was related to staff perceptions of the quality of care delivered in their hospital that is related to patient satisfaction [1]. The second indicator is the Friends and Family Test (FFT) that is routinely used in both staff and patient surveys as a composite measure of patient experience [9]. The FFT is based on the Net Promoter Score [14] that is widely used to measure customer loyalty. The National Health Service (NHS) in the United Kingdom (UK) has included the FFT on the National Staff Survey since 2009 [15]. In Australia, the People Matters Survey administered by the Victorian Public Sector Commission has included the FFT since 2012 [16].

A third variable was staff perceptions of their hospital as a good place to work. Similar to the Friends and Family Test described above, health care staff engagement surveys often include the question “I would recommend my organization as a good place to work” as a summary employee satisfaction indicator [17].

### Employee attitudes

The study included five independent variables derived from other hospital studies: high performance work systems, empowerment, psychological safety, job satisfaction and affective commitment. Each is discussed below. High performance work systems (HPWS) have been identified as critical bundles of management practices that are positively associated with organizational performance in healthcare [18]. High performance work systems influence and align employees’ attitudes and behaviours with the strategic goals of the organization and thereby increase employee commitment and subsequently organizational performance [19]. More specifically, theorists argue the relationship between management systems and organizational outcomes is through influence on individual employee abilities, motivations and job opportunities and through their impact on the collective organization, capabilities and attitudinal climates in which individual perceptions and actions are embedded [20, 21]. Further, Zacharatos et al. [22] argue that high performance work systems concentrate on empowering employees through increased information flows and devolution of decision making to increase employee productivity.

While there has been some debate about the ‘right’ composition of the HPWS bundle [23], it is widely accepted that multiple management practices are involved, and that these practices are mutually reinforcing [5, 20]. As such it is theoretically appropriate to consider HPWS as a single system with a unitary index [24] p.63, and this study used the 15-item HPWS construct developed by Jensen, Patel, and Messersmith [25]. One additional item was added to the validated scale to distinguish between communication within the unit and communication between units.

With the establishment of the HPWS organizational performance link, the science has evolved to identify the pathways through which HPWS may influence the quality of patient care [26]. For example, in a study of nurses, Leggat et al. [1] found that psychological empowerment fully mediated, and job satisfaction moderated, the relationship between HPWS and perceptions of quality of patient care. Similarly, psychological empowerment and affective commitment mediated the relationship between aspects of HPWS and the quality of patient care as perceived by Chinese doctors [2] and relational coordination mediated between HPWS and hospital outcomes [27]. These studies suggest that staff who perceive that their organization has HPWS in place are more likely to rate the quality of care delivered as higher than those staff who do not see evidence of HPWS, with the pathway from HPWS involving positive staff attitudes in relation to empowerment, job satisfaction, relationships among staff and commitment to the organization.

Building upon these previous studies, this study included the variables of psychological empowerment, psychological safety, job satisfaction and affective commitment. Psychological empowerment is defined as a process of enhancing feelings of self-efficacy among employees [28]. Empowerment encourages workers to think for themselves about the requirements of their job, develop meaning for the tasks they are assigned and to enhance their competency levels [29]. Hospital studies have found psychological empowerment to be an important antecedent of quality patient care [3, 30, 31].

Various studies have identified the importance of social relationships [13] and relational coordination [27] on the HPWS - quality of care relationship. In this study we included psychological safety as the measure of staff relationships. Psychological safety is defined as the tacit shared belief among team members that the team environment enables safe interpersonal relationships [32]. Psychological safety was found to be an important relational construct in enhancing team learning among health care organizations [32]. Previous study has illustrated that these social relationships do not have a direct link with performance outcomes, such as perceived quality of care, but have a direct link with empowerment [13, 33].

In the USA, Harmon et al. [18] and Dill and colleagues [34] found a relationship between HPWS and employee and job satisfaction among health care workers. In Australia, Leggat et al. [1] found that job satisfaction moderated the relationship between HPWS and perceived quality of care among nurses. HPWS was found to have a significant positive relationship with perceptions of the quality of patient care among those nurses with higher levels of job satisfaction. Recent research has demonstrated a positive association between HPWS and job satisfaction [35].

**Fig. 1:**
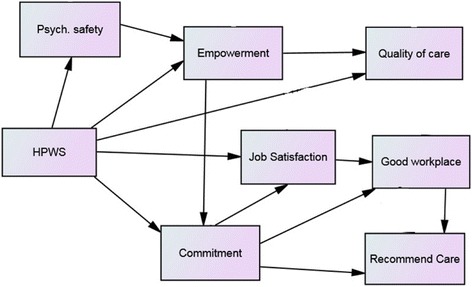
Conceptual model

Considering the research outlined above we proposed three hypotheses that are illustrated in the Fig. 1 framework model.

`

#### *Hypothesis 1:*

*These data can be adequately modelled by a theory-based structural equation model.*

*Hypothesis 2:*

- *High-performing work systems are directly associated with perceived quality of patient care, measured as patient satisfaction (Quality of care) and patient experience (Recommend care).*
- *The relationship between HWPS and quality of patient measured as patient satisfaction is mediated by psychological empowerment.*
- *Psychological safety mediates the relationship between HPWS and empowerment.*

*Hypothesis 3:*

- *The relationship between HPWS and staff perceptions on their organization as a good place to work (Good workplace) is mediated by job satisfaction.*
- *Affective commitment mediates the relationship between HPWS and job satisfaction.*
- *Affective commitment mediates the relationship between HPWS and perceptions of patient experience.*
- *Employees who perceive that their organization is a good place to work will also perceive that it is a good place for friends and family to receive treatment.*

## Methods

A stratified random sample of all departments of 1,000 staff employed by a large multi-campus integrated metropolitan hospital in Australia was invited to participate in a survey designed to explore staff opinions about the care provided by the health service. The sample was segmented to ensure adequate representation of all types of staff employed by the organization. Letters from the Chief Executive Officer, with a hard copy questionnaire, were mailed to the staff member’s home address asking staff to anonymously volunteer to complete the questionnaire. Respondents were also given the option of completing the survey online and were provided with the online survey link. The survey was open from 23 April until 22 May 2014. The survey was anonymous, as staff were not required to include any identifying information and there was no way to identify participants through the online data collection software. The study received ethics approval from the La Trobe University Faculty of Health Science Human Research Ethics Committee in 2014 FHEC14/029. Completion of the study in writing or online was considered to be written consent for participation.

### Measures

The survey (Additional file 1) included five validated scales for the independent variables. These included psychological empowerment [36], job satisfaction [37], organizational commitment [38], employee-level high performance work systems [25] and psychological safety [32]. All scales contained a five-point Likert scale. HPWS was measured with a 16 item scale with Cronbach’s alpha of .872. All 16 items were retained and explored standard management items, such as access to training, communication, performance feedback and appraisal and team working. Empowerment was measured with the 12 item psychological empowerment scale that includes competence, impact, meaning and self-determination. Sample items included, “The work I do is very important to me” (meaning); “I am confident about my ability to do my job” (competence), “I have a great deal of control over what happens in my job” (autonomy), and “My impact on what happens in my job is large” (impact). These items were completed by 243 respondents, the Cronbach’s alpha was .881 and all items were retained.

Job satisfaction was measured with a six item scale with negative items reversed. The questions explored general satisfaction, as well as intent to leave the hospital. These items were completed by 248 respondents, the Cronbach’s alpha was .929 and all six items were retained. Organizational commitment explored attachment to the hospital as a place of employment and was measured with an eight item scale, with negative items reversed. Cronbach’s alpha was .843, with 245 respondents and all nine items were retained. Psychological safety was measured with a seven item scale, with negative items reversed. These items were completed by 246 respondents, the Cronbach’s alpha was .820 and all seven items were retained. Example questions included “If you make a mistake in my unit, it is held against you” and “No one on my unit would deliberately act in a way that undermines my efforts”.

Perceived quality of care was a dependent variable, with two validated measures. The first indicator measured staff perceptions of patient satisfaction with regards to the quality of care delivered within the hospital [1, 12]. This quality of care scale was adapted from the State-wide patient satisfaction questionnaire, and includes items that are seen to be important to patients in assessing the quality of care they have received. This includes perceptions of courtesy, helpfulness, responsiveness and willingness to listen, the provision of information by staff, communication among staff members, safety, privacy, and respect for the patients. Staff were asked to complete a 5-point Likert scale for questions such as: ‘I am responsive to the needs of patients’, ‘I help to relieve the pain of patients’ and ‘I give patients the opportunity to ask questions about their condition or treatment’. The Cronbach’s alpha was .943. All 16 items were retained. This scale was abbreviated as ‘Quality of care’ in the model.

The second was the Friends and Family Test. In the State of Victoria in Australia the FFT question is worded as “I would recommend a friend or relative to be treated as a patient here” [15] and is seen as a useful measure of staff perceptions of the patient experience in their organization. This measure was abbreviated as ‘Recommend care’ in the model. Similarly, respondents indicated whether “I would recommend my organization as a good place to work”, abbreviated as ‘Good workplace’ in the model. In addition there were three demographic tick box questions for sex, tenure at the hospital and discipline.

### Analysis

There were less than 5% missing data and MAR testing revealed data were missing at random. The missing data therefore were imputed using the EM procedure. The results were compared before and after imputation and no major differences were found in the results. Therefore the imputed database used for the final analysis included 251 participants. The responses were analysed using *t*-tests and one-way ANOVA for differences in mean responses related to sex, tenure and discipline of the respondent. No significant differences were observed by sex or tenure. There was one effect for respondent discipline with management and support services staff reporting higher affective commitment than the other disciplines (F 2.29 *p*=0.03).

AMOS (v 20) was used for the structural equation modelling. The fit indices used included the root mean square error of approximation (RMSEA), Tucker-Lewis Index (TLI), Comparative Fit Index (CFI), and Akaike Information Criterion (AIC) [39]. RMSEA values of less than .08 represent the marginal fit, while RMSEA values of less than .05 demonstrate a good fit to the model [40]. TLI and CFI values of greater than 0.90 and 0.95 [41] are considered as marginal and good fits, respectively. The Akaike Information Criterion (AIC) is a comparative measure of fit. AIC can be used meaningfully when more than two models are compared with each other, and a smaller AIC value suggests a superior fit [39]. The chi-square test goodness of fit test was also reported as a conventional, commonly reported measure of absolute fit in the literature. Since the chi square is highly dependent on sample size, the relative chi square (CMIN/DF) was used as a measure of model fit. A value of less than 3 represents acceptable fit [42].

### Test for method effects

To check for possible common method variance (CMV), the Harman’s one-factor test was used to investigate if CMV was an issue in this study. Using this procedure, all of the variables in the study loaded into an exploratory factor analysis using the unrotated factor solution. If one general factor accounts for the majority of the covariance among the measures, then it can be argued that common method variance exists [43]. Based on this procedure the common variance extracted in this study was not a pervasive issue since it accounted for only 25% of the total variance extracted.

## Results

Eleven of the mailed surveys were returned to sender unopened, suggesting a final distribution of 989. A total of 258 individuals completed the questionnaire (26% response rate), which is comparable to other published large scale health service staff surveys see, for example, [44, 45]. To check for responder bias, the distribution of the sample characteristics of the respondents were compared to 455 respondents of a shorter survey on the same topic. There were no significant differences found. Out of these 258 individuals, 242 respondents indicated their sex, with 195 females (75.63%) and 47 males (18.27%). The majority of the respondents were nurses (50%) followed by allied health professionals and doctors at about 11% each, administrative and clerical staff (10%) and mangers (3%). The remaining 15% comprised corporate and support services staff and 4% of staff whose role did not fit in any of these categories. The reported tenure of the respondents was 31 (13%) with less than one year, 59 (24%) with one to four years, 62 (25%) with five to nine years, 45 (18%) with 10 to 14 years and 50 (20%) with 15 or more years working at this organization. This distribution is consistent with the staff distribution and tenure throughout the organization.

### Model Fit Evaluation

The model was tested using a multivariate statistical model using Maximum Likelihood (ML) estimation, assuming multivariate normality. The univariate normality assessment showed relatively normal distribution of the majority of the variables (Kurtosis less than 1), with the exception of the ‘quality of care’ variable (Kurtosis of 9.40) indicating moderate non-normality. However, Mardia’s Multivariate Kurtosis coefficient (Mardia's normalized coefficient = 11.09) meets the multivariate normality assumptions by comparing it with the formula p(p+2) where p is the number of observed variables in the model [46]. Since the Mardia’s coefficient is lower than the value obtained from the above formula, then the data meets the multivariate normality assumption.

**Fig. 2:**
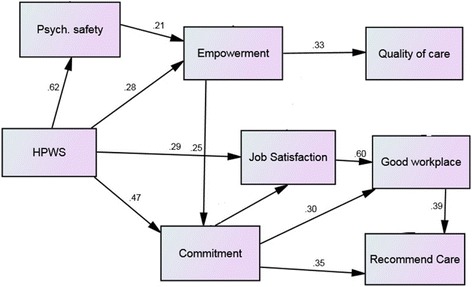
Final model of the study

Although the preliminary analysis demonstrated a good model fit (*χ* ^2^/*df*=2.19, TLI=.95, CFI=.98, RMSEA=.07), no significant direct path from HPWS to the perceived quality of patient care was found. The second model (Fig. 2), where this direct relationship was removed demonstrated a great model fit (*χ* ^2^/*df* =2.05, TLI=.97, CFI=.98, RMSEA=.06). Comparing the AIC of the modified model (AIC= 88.81) with the first model (AIC=90.98) suggested a smaller AIC for the model, also demonstrating the superiority of the second model (Table 1).

**Table 1:** Model fit statistics

Model	χ2/df	RMSEA	TLI	CFI	AIC
1. The proposed model	2.19	.07	.95	.98	90.98
2. The modified model	2.05	.06	.97	.98	88.81

Note: RMSEA= Root Mean Square Error of Approximation; The Tucker-Lewis Index (TLI); Comparative Fit Index (CFI); AIC = Akaike Information Criterion

The preferred model suggested that there were different paths to the perceived quality of care and perceived patient experience indicators for this group of staff. It is evident in Fig. 2 that in the first path that the relationship between HPWS and perceived quality of care is significantly mediated by empowerment. In addition, psychological empowerment was shown to have positive effects on affective commitment and psychological safety mediated the relationship between HPWS and empowerment.

The second path highlighted a relationship between HPWS and the outcome measures of ‘I would recommend my organization as a good place to work’ and the Friends and Family Test (patient experience) showing that job satisfaction mediated the relationship between staff perceptions of HPWS and their perceptions on their organization as a good place to work and affective commitment mediated the relationship between HPWS and job satisfaction and between staff perceptions of HPWS and both outcome measures.

## Discussion

Our results provide insight into how the relationship between people management practices, measured as HPWS, and staff perceptions of the quality of care that they deliver is enacted. In this study HPWS comprised the people management practices of opportunities for training and development, selective hiring, teams and decentralized decision making, job security, information sharing, transformational leadership, and performance feedback. In this multi-site hospital we identified one path from staff perceptions of HPWS to the perceived quality of care indicator associated with patient satisfaction items. This path was mediated by empowerment, and the relationship between HPWS and empowerment was mediated by psychological safety. The second path from staff perceptions of HPWS to the Friends and Family Test, summarising perceived patient experience, was mediated by affective commitment and was directly related to staff perceptions about their workplace being a good place to work.

### The path to patient satisfaction perceived quality of care

The first path, from staff perceptions of HPWS to perceived quality of care, suggests that staff who perceived that the part of the organization in which they worked had good management in place, and who reported higher levels of psychological safety and empowerment, also indicated that they perceived that their patients would have higher satisfaction with the care provided. These findings expand upon the contention that good management is fundamental to patient experience, safety and quality of care [47] by outlining the staff attitudes that need to be fostered by management to contribute to quality of care. All types of health care staff recognise HPWS in their organization and the findings transcend national cultures [1, 2, 4, 52]. Importantly, our study included all hospital employees, and we were able to extend the findings of other studies that found that empowerment is a mediator between management practice and perceived quality of care of nursing staff [1, 26] to hospital staff more generally. Clearly, quality of patient care is a critical part of the cultural values of all hospital workers, not only the domain of clinicians [12, 48], and our results show that similar relationships exist among the perceptions of various types of hospital staff.

Our results further support the evidence of a positive link between reported staff empowerment and quality of care [46, 47], by demonstrating that psychological empowerment is positively associated with perceptions of quality of care. Lack of empowerment of staff has been identified as a substantial barrier to improvement of quality and safety [49], which has led to the design of quality and safety improvement processes that address staff needs for empowerment [50, 51]. Our findings explain why empowerment is linked with success in quality and safety improvement. Good management is necessary but not sufficient for quality of care delivery and staff must feel both empowered and psychologically safe in their work roles.

Psychological safety has been identified as an important teamwork variable in improving quality of care [32], especially as an antecedent to staff ‘speaking up’ about safety issues [52]. Our model clearly illustrates the strong relationship between staff perceiving that their work unit provides them with psychological safety and their feelings of empowerment in their role. Recent studies have identified the complexities of successful implementation of quality and safety initiatives and stress the need for implementation that addresses psychological safety [53], in addition to empowerment. While we cannot claim causality, there is mounting evidence that enhancing quality and safety requires effective management practice that promotes staff empowerment in a psychologically safe work environment.

### The path to patient experience perceived quality of care

In the second path the relationship between staff perceptions of HPWS and the outcome variable of staff recommending their organization as a good place to work was mediated by job satisfaction, and the relationship between job satisfaction and a good place to work was partially mediated by affective commitment. In addition, the relationship between staff perceptions of HPWS and the second outcome variable of perceived patient experience (FFT) was mediated by affective commitment. This suggests that the FFT measures an aspect of quality of care that is more related to staff connection with the organization in which they work than with their perceptions of their performance. Staff who perceive HPWS in place and who have a strong emotional relationship with their organization, who believe their organization is a good place to work, will report higher scores on the FFT of patient experience. This supports an earlier study that found that the FFT was not a strong predictor of the actual quality of care provided, as staff were influenced by a range of factors, many of which had little bearing on the patient experience with care [54].

Our findings show that patient satisfaction and patient experience indicators of perceived quality of care are enacted through different organizational and attitudinal paths. While HPWS, or perceptions of good management practice were apparent in both paths, perceptions of patient satisfaction related to quality of care were linked with the psychological constructs of empowerment and psychological safety. That is, the staff were more likely to evaluate the patient satisfaction quality of care indicator in relation to how they perceived their performance on the job. In contrast, the FFT patient experience indicator appears to measure staff attachment and satisfaction with the organization in which they work. We did not find any relationship between perceptions of the quality of care that was delivered and the FFT patient experience indicator.

Interestingly, the managers and support services staff in our sample generally reported higher levels of commitment to the organization than the clinical staff, and therefore may have more positive perceptions about the hospital and patient experience than the clinical staff. However, there were no differences found between clinical and non-clinical staff in relation to the attitudes underpinning quality of care measured as perceived patient satisfaction. These findings suggest that staff perceive the quality of care indicators measured as patient satisfaction and patient experience quite differently and reinforce the dangers of “…ever more centralized, standardized, and unified measures of quality that are common in policy discourse and interventions” [55] p. 186. Our findings suggest that it is important to continue to use a variety of quality and safety measures that are meaningful to both hospital and patients staff, as staff perceive the organizational and attitudinal factors contributing to quality and safety indicators to be different for different indicators.

### Limitations

This was a cross-sectional study and while the final pathway received good support, the data do not confirm causality. Despite the lack of common method variance, the generalisability of the findings may be limited due to the self-selected study population in one Australian hospital, the relatively low response rate and the use of self-reported data. In addition, we used single items in the FFT test and the organization as a good place to work, which does not add strength to the measurements, but is consistent with current practice in system and organizational surveys. Further study to specifically analyse the pathways for different types of staff would enable extension of these results.

## Conclusions

Responding to the limited evidence on effective roles of hospital managers in quality and safety [56], this study provides empirical evidence of the need for managers to embrace identified good people management practices [57], including ensuring opportunities for training and development, selective hiring, promoting teamwork and decentralized decision making, information sharing, transformational leadership, and providing performance feedback, to embed psychological safety and empower staff. Our findings illustrated that there is not a direct link between staff perceptions of HPWS and perceived quality of care, but that it is enacted through psychological safety and empowerment. In comparison, the Friends and Family Test question that asks about recommending treatment to friends and family, while also associated with staff perceptions of HPWS, was mediated by affective commitment and strongly linked to the perception of staff as the organization being a good place to work. This study highlights the different pathways to perceived quality of care and perceived patient experience among hospital staff.

## Additional file

Additional file 1: Questionnaire. (DOCX 27 kb)

## Abbreviations

AIC: Akaike information criterion
CFI: Comparative fit index
CMV: Common method variance
FFT: Friends and Family Test
HPWS: High performance work systems
RMSEA: Root mean square error of approximation
SEM: Structured equation model
TLI: Tucker-Lewis index
