# Supplementary material for: A path analysis study of factors influencing hospital staff perceptions of quality of care factors associated with patient satisfaction and patient experience
Source: BMC Health Serv Res. 2017 Nov 16;17:739. doi: 10.1186/s12913-017-2718-x (PMC5693360; doi:10.1186/s12913-017-2718-x)
Supplement: Additional file 1: — Questionnaire. (DOCX 27 kb) [file 12913_2017_2718_MOESM1_ESM.docx]

Appendix A Questionnaire

**Your feelings about your job at ___________:**

Please respond to each of the following statements by clicking the number than is most relevant to you.

| **SD** = Strongly Disagree  (1) | **D** = Disagree  (2) | **N** = Neither Agree nor Disagree  (3) | **A** = Agree  (4) | **SA** = Strongly Agree  (5) |
| --- | --- | --- | --- | --- |

**Empowerment**

| **SD** | **D** | **N** | **A** | **SA** |  |
| --- | --- | --- | --- | --- | --- |
| The work I do is very important to me | 1 | 2 | 3 | 4 | 5 |
| My job activities are personally meaningful to me | 1 | 2 | 3 | 4 | 5 |
| The work I do is meaningful to me | 1 | 2 | 3 | 4 | 5 |
| I am confident about my ability to do my job | 1 | 2 | 3 | 4 | 5 |
| I am self-assured about my capabilities to perform my work activities | 1 | 2 | 3 | 4 | 5 |
| I have mastered the skills necessary for my job | 1 | 2 | 3 | 4 | 5 |
| I have significant autonomy in determining how I do my job | 1 | 2 | 3 | 4 | 5 |
| I can decide on my own how to go about doing my work | 1 | 2 | 3 | 4 | 5 |
| I have considerable opportunity for independence and freedom in how I do my job | 1 | 2 | 3 | 4 | 5 |
| My impact on what happens in this unit is large | 1 | 2 | 3 | 4 | 5 |
| I have a great deal of control over what happens in this unit | 1 | 2 | 3 | 4 | 5 |
| I have significant influence over what happens in this unit | 1 | 2 | 3 | 4 | 5 |

**Commitment**

| **SD** | **D** | **N** | **A** | **SA** |  |
| --- | --- | --- | --- | --- | --- |
| I would be very happy to spend the rest of my career with this organisation | 1 | 2 | 3 | 4 | 5 |
| I enjoy discussing my organisation with people outside it | 1 | 2 | 3 | 4 | 5 |
| I really feel that this organisation’s problems are my own | 1 | 2 | 3 | 4 | 5 |
| I think I could become easily attached to another organisation as I am to this one | 1 | 2 | 3 | 4 | 5 |
| I do not feel like “part of the family” at this organisation | 1 | 2 | 3 | 4 | 5 |
| I do not feel “emotionally attached” to this organisation | 1 | 2 | 3 | 4 | 5 |
| This organisation has a great deal of personal meaning to me | 1 | 2 | 3 | 4 | 5 |
| I do not feel a strong sense of belonging to my organisation | 1 | 2 | 3 | 4 | 5 |
| I would recommend a friend or relative to be treated as a patient here | 1 | 2 | 3 | 4 | 5 |

**Why would you recommend treatment at _________________ to your friend or relative?**

**Why would you NOT recommend treatment at ________________ to your friend or relative?**

**Psychological Safety**

| **SD** | **D** | **N** | **A** | **SA** |  |
| --- | --- | --- | --- | --- | --- |
| 1. If you make a mistake in my unit, it is often held against you. | 1 | 2 | 3 | 4 | 5 |
| 2. Members of my unit are able to bring up problems and tough issues. | 1 | 2 | 3 | 4 | 5 |
| 3. People on my unit sometimes reject others for being different. | 1 | 2 | 3 | 4 | 5 |
| 4. It is safe to take a risk on my unit. | 1 | 2 | 3 | 4 | 5 |
| 5. It is difficult to ask other members of my unit for help. | 1 | 2 | 3 | 4 | 5 |
| 6. No one on my unit would deliberately act in a way that undermines my efforts. | 1 | 2 | 3 | 4 | 5 |
| 7. Working with members of my unit, my unique skills and talents are valued and utilised. | 1 | 2 | 3 | 4 | 5 |

**Job Satisfaction**

| **SD** | **D** | **N** | **A** | **SA** |  |
| --- | --- | --- | --- | --- | --- |
| In general, I don’t like my job | 1 | 2 | 3 | 4 | 5 |
| In general I like working here | 1 | 2 | 3 | 4 | 5 |
| I often think of quitting the organisation | 1 | 2 | 3 | 4 | 5 |
| I think of searching for another position with another organisation | 1 | 2 | 3 | 4 | 5 |
| I often think of leaving the organisation within the next year | 1 | 2 | 3 | 4 | 5 |
| All in all, I am satisfied with my job | 1 | 2 | 3 | 4 | 5 |
| I would recommend my organisation as a good place to work | 1 | 2 | 3 | 4 | 5 |

| **SD** = Strongly Disagree  (1) | **D** = Disagree  (2) | **N** = Neither Agree nor Disagree  (3) | **A** = Agree  (4) | **SA** = Strongly Agree  (5) |
| --- | --- | --- | --- | --- |

**Patient Focus**

| **SD** | **D** | **N** | **A** | **SA** | | **N/A** | |
| --- | --- | --- | --- | --- | --- | --- | --- |
| I am courteous to patients | 1 | *2* | *3* | *4* | *5* | *6* | |
| I am responsive to the needs of patients | 1 | 2 | 3 | 4 | 5 | 6 | |
| I respond to patient’s call quickly | 1 | 2 | 3 | 4 | 5 | 6 | |
| I provide good quality of information to the patients about their treatment | 1 | 2 | 3 | 4 | 5 | 6 | |
| I communicate with other clinicians and management about patient’s treatment | 1 | 2 | 3 | 4 | 5 | 6 | |
| I am generally helpful | 1 | 2 | 3 | 4 | 5 | 6 | |
| I help to relieve the pain of patients | 1 | 2 | 3 | 4 | 5 | 6 | |
| I respect my patient’s privacy during their stay | 1 | 2 | 3 | 4 | 5 | 6 | |
| I respect the cultural and/or religious needs of patients | 1 | 2 | 3 | 4 | 5 | 6 | |
| I look after personal safety | 1 | 2 | 3 | 4 | 5 | 6 | |
| I treat patients with respect | 1 | 2 | 3 | 4 | 5 | 6 | |
| I give patients the opportunity to ask questions about their condition or treatment | 1 | 2 | 3 | 4 | 5 | 6 | |
| I always involve patients in decisions about their care | 1 | 2 | 3 | 4 | 5 | 6 | |
| I am willing to listen to patient’s health care problems | 1 | 2 | 3 | 4 | 5 | 6 | |
| I always explain the purposes of medicines to patients | 1 | 2 | 3 | 4 | 5 | 6 | |
| I always explain the possible side-effects of medicines to patients | 1 | 2 | 3 | 4 | 5 | 6 | |

**What would you change to improve health care to patients?**

**High Performance Work Systems**

| **SD** | **D** | **N** | **A** | **SA** |  |
| --- | --- | --- | --- | --- | --- |
| I am provided with sufficient opportunities for training and development. | 1 | 2 | 3 | 4 | 5 |
| I receive the training I need to do my job. | 1 | 2 | 3 | 4 | 5 |
| This department keeps me informed about business issues and about how well it’s doing. | 1 | 2 | 3 | 4 | 5 |
| There is clear status difference between management and staff in my unit. | 1 | 2 | 3 | 4 | 5 |
| Team working is strongly encouraged in my unit. | 1 | 2 | 3 | 4 | 5 |
| A rigorous selection process is used to select new recruits. | 1 | 2 | 3 | 4 | 5 |
| Management involve people when they make decisions that affect them. | 1 | 2 | 3 | 4 | 5 |
| Communication *within* this unit is good. | 1 | 2 | 3 | 4 | 5 |
| Communication *between* units is good. | 1 | 2 | 3 | 4 | 5 |
| I feel my job is secure. | 1 | 2 | 3 | 4 | 5 |
| The rewards I receive are directly related to my performance at work. | 1 | 2 | 3 | 4 | 5 |
| Career management is given a high priority in my unit. | 1 | 2 | 3 | 4 | 5 |
| I have the opportunities I want to be promoted. | 1 | 2 | 3 | 4 | 5 |
| The appraisal system provides me with an accurate assessment of my strengths and weaknesses. | 1 | 2 | 3 | 4 | 5 |
| I am given meaningful feedback regarding my performance at least once a year. | 1 | 2 | 3 | 4 | 5 |

| My knowledge about my job is transferred to senior management levels in the organisation | 1 | 2 | 3 | 4 | 5 |
| --- | --- | --- | --- | --- | --- |

**Leadership**

*My immediate manager:*

| **SD** | **D** | **N** | **A** | **SA** |  |
| --- | --- | --- | --- | --- | --- |
| is focused on doing the right thing as well as on getting results | 1 | 2 | 3 | 4 | 5 |
| encourages employees to look at problems and come up with their own solutions and suggestions | 1 | 2 | 3 | 4 | 5 |
| listens to the concerns of employees | 1 | 2 | 3 | 4 | 5 |
| expresses their confidence that the unit will achieve its goals | 1 | 2 | 3 | 4 | 5 |
| encourages employees to express their ideas and opinions | 1 | 2 | 3 | 4 | 5 |
| provides employees with continuous encouragement | 1 | 2 | 3 | 4 | 5 |
| provides me with motivation | 1 | 2 | 3 | 4 | 5 |

| deserves trust, can be believed and relied upon for his/her word | 1 | 2 | 3 | 4 | 5 |
| --- | --- | --- | --- | --- | --- |
| tends to conceal information from others | 1 | 2 | 3 | 4 | 5 |
| is open in his or her communication with subordinates | 1 | 2 | 3 | 4 | 5 |
| pursues his or her best interests at the expense of others | 1 | 2 | 3 | 4 | 5 |
| acts according to what is right or fair | 1 | 2 | 3 | 4 | 5 |
| is not sincere, is fraudulent | 1 | 2 | 3 | 4 | 5 |
| makes sure that his/her actions are always ethical | 1 | 2 | 3 | 4 | 5 |
| speaks and acts truthfully | 1 | 2 | 3 | 4 | 5 |
| would be personally inclined to use his/her power to help me solve problems in my work | 1 | 2 | 3 | 4 | 5 |
| would bail me out even at his or her own expense, when I really need it | 1 | 2 | 3 | 4 | 5 |
| understands my problems and needs | 1 | 2 | 3 | 4 | 5 |
| recognises my potential | 1 | 2 | 3 | 4 | 5 |
| has enough confidence in me that he/she would defend and justify my decisions if I were not present | 1 | 2 | 3 | 4 | 5 |
| I usually know where I stand with my immediate manager | 1 | 2 | 3 | 4 | 5 |
| I have excellent working relationship with my immediate manager | 1 | 2 | 3 | 4 | 5 |
